# Naltrexone treatment for prolonged grief disorder: study protocol for a randomized, triple-blinded, placebo-controlled trial

**DOI:** 10.1186/s13063-021-05044-8

**Published:** 2021-02-01

**Authors:** James Gang, James Kocsis, Jonathan Avery, Paul K. Maciejewski, Holly G. Prigerson

**Affiliations:** 1 Center for Research on End-of-Life Care, Weill Cornell Medicine, New York, NY USA; 2 Department of Psychiatry, Weill Cornell Medicine, New York, NY USA; 3 Department of Medicine, Weill Cornell Medicine, New York, NY USA; 4 Department of Radiology, Weill Cornell Medicine, New York, NY USA

**Keywords:** Prolonged grief disorder, Naltrexone, Pharmacological treatment, Randomized control trial

## Abstract

**Background:**

There is a lack of effective pharmacotherapy for prolonged grief disorder (PGD). Evidence suggests that the neurobiology of PGD involves the same circuitry as the reward pathway. Based upon this evidence, we hypothesize that PGD can be conceptualized as a disorder of addiction and therefore could benefit from being treated with medications that are currently used to treat such disorders. One such medication is naltrexone, which is currently used to treat alcohol and opioid dependence. Oral naltrexone was chosen for its mechanism of action, safety, and convenience. The primary aim of this study is to establish the efficacy of using oral naltrexone as a pharmacological treatment for PGD. Specifically, we hypothesize that participants receiving naltrexone will demonstrate reduced PGD symptoms when compared to placebo.

**Methods/design:**

This is a randomized, placebo-controlled, triple-blinded (to healthcare professionals/study staff, participants, and data analysts) study in which we propose to enroll 48 participants who meet criteria for Prolonged Grief Disorder (PGD). Participants will be randomly assigned to the naltrexone 50 mg oral arm or placebo arm; medications will be over-encapsulated to appear identical. Participants will take their assigned medication for 8 weeks, with clinic visits every 4 weeks to assess symptom severity, social closeness, and adverse reactions. Weekly surveys of Prolonged Grief-13-Revised (PG-13-R) will be used to relate naltrexone use to changes in PGD symptom severity. Follow-up 4 weeks after their last visit will assess the longevity of treatment, as well as any lingering adverse reactions.

**Discussion:**

This study is the first to investigate the use of oral naltrexone as pharmacological treatment for PGD. The acute and debilitating nature of the disorder, in addition to the increased risk of comorbidities, highlights the need for pharmacological treatment like naltrexone that can act more rapidly, may help those for whom psychotherapy may not be effective, and/or may augment psychotherapy to promote PGD symptom grief resolution.

**Trial registration:**

ClinicalTrials.govNCT04547985. Registered on 8/31/2020.

## Administrative information

The order of the items has been modified to group similar items (see http://www.equator-network.org/reporting-guidelines/spirit-2013-statement-defining-standard-protocol-items-for-clinical-trials/).

**Table:** 

Title {1}	Naltrexone treatment for prolonged grief disorder: study protocol for a randomized, triple-blinded, placebo-controlled trial
Trial registration {2a and 2b}	ClinicalTrials.gov, NCT04547985. Registered on 8/31/2020, https://clinicaltrials.gov/ct2/show/NCT04547985.
Protocol version {3}	10/08/20; v2.2
Funding {4}	This work is supported by the National Cancer Institute (NCI) (grant number CA197730 and CA218313) and the Clinical Translational Science Center (CTSC), National Center for Advancing Translational Sciences (NCATS) grant TR002384.
Author details {5a}	JG, PM, HGP are affiliated with the Center for Research on End-of-Life Care at Weill Cornell Medicine. PA and JK are affiliated with the Department of Psychiatry at Weill Cornell medicine.
Name and contact information for the trial sponsor {5b}	Weill Medical College of Cornell University 1300 York Avenue, Box 305 New York, NY 10065 Phone: (646) 962-8215
Role of sponsor {5c}	Funders played no role in the design of this study and will not have any role during its execution, analyses, interpretation of the data, or decision to submit results.

## Introduction

### Background and rationale {6a}

The recent inclusion of prolonged grief disorder (PGD) in the ICD-11 [1, 2] and the DSM [3, 4] has been a catalyst for research to advance understanding and treatment of this newly recognized mental disorder. PGD, a maladaptive response to the death of a loved one, is characterized by persistent yearning for the deceased and disabling symptoms such as emotional numbness, a sense of disbelief about the death, identity disruption, and an inability to move on and find meaning in life in the absence of the deceased [5]. When these symptoms are functionally impairing and severe at 12 months post-loss, a person can now be diagnosed as meeting criteria for PGD [5]. The 7–10% of bereaving people estimated to be meet criteria for PGD have been shown to be at increased risk of medical conditions and diseases (e.g., hypertension, immunological dysfunction, cancer), physical and mental impairments, reduced quality of life, suicidal ideation (SI) and attempts, emergency room visits, and multiple nights spent in the hospital [6–9]. The severe and substantial risks posed by a PGD diagnosis highlight the need to investigate ways to help those suffering from this new mental disorder.

While psychotherapy tailored specifically for PGD has proven effective in reducing PGD symptom severity in some people [10, 11], the acute and debilitating nature of the disorder highlights the need for pharmacological treatment that can act more rapidly, may help those for whom psychotherapy may not be effective, and/or may augment psychotherapy to promote PGD symptom grief resolution. Current evidence on pharmacological intervention for PGD is scarce. One barrier to testing pharmacologic agents has been the absence of PGD as a recognized mental disorder. Based on the clinical rationale that PGD exhibits similar symptoms to major depressive disorder (MDD) and post-traumatic stress disorder (PTSD), selective serotonin reuptake inhibitors (SSRIs) have been explored in three open-label trials and one case series. While these studies demonstrate moderate effectiveness in reducing grief symptoms, the results are confounded by high rates of comorbid mental disorders, and the scientific rigor has been undermined by lack of randomization and blinding, small sample sizes, and low levels of statistical power [12]. The other medications trialed have included tricyclic antidepressants (TCAs) and benzodiazepines, both of which have not proven effective for the reduction of symptoms of PGD [13, 14].

We believe that treating PGD as a disorder akin to MDD or PTSD is misguided, for two main reasons. The first reason is that PGD has been shown to demonstrate a distinct neural profile when compared to MDD or PTSD [15]. In contrast to conceptualizing PGD as a mood disorder such as MDD or a stress response syndrome such as PTSD, there is emerging evidence to suggest that PGD may be conceptualized as a reward dysfunction disorder, with the deceased person as the rewarding stimulus for whom the bereaved person yearns [16]. PGD, as noted above, is at its core a disorder of attachment and a craving and yearning for the deceased from whom they are separated (resulting in significant separation distress). In PGD, the primary gateway symptom required for diagnosis is yearning: persistent longing, pining for, or preoccupation with, the deceased. In this way, patients with PGD continue to “crave” their loved ones after they have died, due to the positive reinforcement provided by their memories of loved ones. The absence of the deceased creates a feeling of withdrawal—missing the person, feeling focused on his or her absence, feeling like life is not pleasurable and actually painful without the deceased, sorrow, resentment and agitation at being denied the support and security, meaning, and identity the deceased provided.

A second reason for finding an alternative to SSRIs for the treatment of PGD is that they take weeks to months to achieve full efficacy. Persons suffering from PGD would benefit from faster acting interventions, especially given significant suicide attempt risk of those meeting criteria for PGD [17]. A delay in resolution of PGD symptoms would protract the period of risk for suicidal thoughts and behaviors with which PGD is associated [4, 17].

Neurobiologically, numerous studies suggest that there are associations between symptoms of PGD and the reward pathway, which is the same pathway primarily responsible for addiction [16]. The reward pathway refers to a group of interconnected structures in the brain that uses dopamine as a signal to modulate the experience of reward. Some pertinent examples of enhanced activation in brain structures in PGD that are also important to the reward pathway include the nucleus accumbens [18] and orbitofrontal cortex [15].

Based on evidence supporting an association between PGD and neurobiological correlates of reward and addiction, we hypothesize that treatments for addiction might prove successful where those of MDD and PTSD have failed. Currently, disorders of addiction are treated with a wide variety of medications based upon the substance being abused, including bupropion and varenicline for tobacco use; naltrexone and acamprosate for alcohol use; methadone and buprenorphine for opioid use [19]. Of these, we have opted to trial naltrexone to treat PGD because of its mechanism of action, effects on social attachment, and side-effect profile.

Mechanistically, naltrexone is a competitive antagonist of opioid receptors. These receptors, when bound by endogenous opioids released in response to rewarding stimuli, normally cause the subsequent release of dopamine in the reward pathway. Therefore, naltrexone may be an appropriate choice to treat PGD, as its mechanism of action directly affects the reward pathway; by blocking opioid receptors, release of dopamine in the reward pathway is inhibited. It also has two practical advantages for patients. First, it can be administered orally (daily) or intramuscularly (monthly). Second, cost of oral naltrexone is not prohibitively high and is often covered by insurance [20].

Symptomatically, naltrexone targets two crucial aspects of PGD. First, assuming that having and maintaining social connections is vital to human functioning, the opioid theory of social attachment suggests that endogenous opioids are released during these experiences of social bonding, which then underlie the pleasant feelings of social bonding to positively reinforce the formation of such bonds. Based on this theory, studies have shown that naltrexone reduces feelings of social connection, especially to one’s closest others [21, 22]. Reduced positive associations with significant others, especially the deceased, may make bereavement feel less lonely and isolated while diminishing the reward derived from reminiscing about the deceased. Second, meta-analysis confirms that naltrexone reduces craving in patients with alcohol dependence [23]; in PGD, craving the deceased is the core symptom [5]. Thus, naltrexone may reduce the craving for the deceased and thereby severity of PGD. Given these findings, we predict that naltrexone will provide a pharmacological way to dampen the benefits of social bonding while reducing the yearning or craving for the deceased loved one, which would reduce the severity of PGD.

With respect to side effects, naltrexone is well-tolerated and may be better tolerated than TCAs or SSRIs. The most common side effects of naltrexone include nausea, vomiting, abdominal pain, headache, and fatigue. The most severe side effect warranting an FDA black-box warning is hepatotoxicity; in the COMBINE study, elevations in liver function tests (LFTs) were seen in only 11 of 614 participants, with most normalizing after cessation and no serious long-term consequences [24]. In addition, although naltrexone can cause sudden and severe withdrawal in opiate users, we would not trial naltrexone in bereaved individuals who are opiate users.

### Objectives {7}

The primary objective is to determine the efficacy of naltrexone in reducing PGD symptoms compared to placebo.

#### Secondary objective

The secondary objective is to evaluate the effects of naltrexone on social closeness with both the deceased (the bereaved person) and the living (surviving others and social network).

#### Exploratory objectives

- To evaluate associations between naltrexone and social integration and social connectedness.
- To evaluate associations between naltrexone and suicidal thoughts and behaviors.
- To evaluate associations between naltrexone and health behaviors (e.g., alcohol and tobacco use, sleep, food consumption).
- To evaluate associations between outcomes of COVID-19-related deaths versus other causes of death.

### Trial design {8}

This study is designed as a randomized, placebo-controlled, triple-blinded (to healthcare professionals, participants, and data analysts), stage IV, single-site superiority trial with two parallel groups.

## Methods: participants, interventions and outcomes

### Study setting {9}

The study will take place at NewYork Presbyterian – Weill Cornell Medicine (NYP-WCM) in New York, NY, USA.

### Eligibility criteria {10}

#### Inclusion criteria

1. 18 years of age or older and younger than 90 years of age.
2. Lives within a reasonable distance from NYP-WCM for convenient clinic visits.
3. Can speak, read, and write English proficiently.
4. Meet diagnostic criteria for PGD based on/consistent with the DSM criteria (American Psychiatric Association, n.d.), which includes:
  - Death of a person the bereaved survivor considered a significant other.
  - Since the death, there has been a grief response characterized by intense yearning/longing for the deceased person or a preoccupation with thoughts or memories of the deceased person present nearly every day for the last month.
  - As a result of the death, at least 3 of the following symptoms have been experienced nearly every day, for at least the last month: identity disruption (e.g., feeling as though part of oneself has died); marked sense of disbelief about the death; avoidance of reminders that the person is dead; intense emotional pain (e.g., anger, bitterness, sorrow) related to the death; difficulty moving on with life (e.g., problems engaging with friends, pursuing interests, planning for the future); emotional numbness; feeling that life is meaningless; intense loneliness (i.e., feeling alone or detached from others).
5. If female, must agree to use a method of contraception and be willing and able to continue contraception during the first 8 weeks of the study while taking the study drug. Female patients who are planning to use oral hormonal contraception during this time must have initiated it at least 2 months prior to the baseline visit.
6. If male, must agree to use a method of contraception and be willing and able to continue contraception during the first 8 weeks of the study while taking the study drug.

#### Exclusion criteria

1. Having recently started taking/prescribed medications for any psychiatric illness (e.g., SSRIs for MDD) within the past 3 months; participants who have been taking this medication for longer than 3 months can be included.
2. Having recently started psychotherapy for any psychiatric illness within the past 3 months; participants who have been receiving psychotherapy for longer than 3 months can be included.
3. Prior history of recently active (e.g., within the past 3 months) opioid dependence.
4. Current prescription, non-prescription, or illicit opioid use (i.e., acute use within the past 14 days or chronic use within the last 30 days), including opioid antagonists for alcohol or opioid dependence, all opioid analgesics, certain cough and cold remedies (e.g., codeine), and certain anti-diarrheal preparations (e.g., loperamide).
5. Possible future use of opioids during the study (e.g., for surgery).
6. Current use of leflunomide (Arava), droperidol (Droleptan), diazepam (Valium), thioridazine (Mellaril, Novoridazine, Thioril), or any other clinically relevant medication that has potential to cause liver injury with concurrent use of naltrexone.
7. Currently pregnant, lactating, or planning to become pregnant during the study.
8. Active hepatitis or liver disease.
9. Alanine aminotransferase (ALT) or aspartate aminotransferase (AST) levels more than one standard deviation above the upper limit of normal on initial laboratory examination.
10. Screen positive for active suicidal thoughts or behaviors.

### Who will take informed consent? {26a}

Written informed consent will be obtained from participants by trained research assistants at the first visit. During the informed consent process, the study staff obtaining consent will review the study in detail allowing for the participant to interject with questions at any time during the discussion. The main points that will be addressed by the study personnel obtaining informed consent include explaining why the current research study is being conducted, the sources of funding for the project and what purpose the proposed research serves. Additionally, the subject will be made aware of who is responsible for conducting the research and how subjects are selected for participation in the research.

The study staff will explain in explicit detail what will be asked of the participant if he/she agrees to participate in the study and estimate the potential time commitment involved. Participant will know what his/her responsibilities will entail, what risks or benefits may be involved, and what potential costs could be incurred should they agree to participate. Study staff will underscore the importance placed upon maintaining participant confidentiality, as well as participant’s rights while involved in the study, which includes the right to withdraw participation at any time during the research since their participation is entirely voluntary in nature.

The informed consent form will also contain a section dedicated to explaining what constitutes protected health information and how this information remains confidential per HIPAA (Health Insurance Portability and Accountability Act) guidelines. Finally, the informed consent form will provide contact information for both the principal investigators as well as the Office for the Protection of Research Subjects. All informed consent processes will adhere to the policies set forth by the Institutional Review Board.

### Additional consent provisions for collection and use of participant data and biological specimens in ancillary studies, if applicable. {26b}

Participant data and biological specimens will not be used in ancillary studies.

## Interventions

### Explanation for the choice of comparators {6b}

Placebo was chosen as control in this study, for two reasons: (1) the presence of a placebo mitigates the placebo effect, and (2) as a new disorder, there is no standard of care treatment for PGD; thus, most patients in real life are, by default, not receiving treatment at all [10].

### Intervention description {11a}

Naltrexone HCl 50 mg is an oral medication in tablet form purchased from an FDA-licensed drug wholesaler. Naltrexone will be compounded and over-encapsulated into a gelatin capsule stable for 6 months at room temperature with instruction to be taken as is PO with a glass of water. Placebo, composed of a filler material (e.g., lactose or methyl cellulose), will be identical in appearance to naltrexone via over-encapsulation. The tablets are packaged into white, high-density polyethylene bottles fitted with child-resistant closures with foil induction seals, each containing 56 tablets. Each bottle also contains a polyester coil as bottle filler. Bottles will be labeled as such that only the FDA-licensed drug wholesaler will know which ones contain naltrexone and which ones contain placebo; this information will only be shared with the principal investigator (PI).

All participants will take their assigned medication daily for 8 weeks, starting the day after their first clinic visit. This medication will be dispensed once during this first clinic visit. For naltrexone, the daily dose will be 50 mg. Participants will be advised to take it around lunchtime, but this is not a strict requirement; naltrexone can be taken with or without food. There is no anticipated adverse reaction from stopping naltrexone at any point in the study; therefore, there is no maximum or minimum duration. If participants miss a dose, they can resume the normal schedule the next day; there is no need to compensate for any missed doses.

### Criteria for discontinuing or modifying allocated interventions {11b}

An investigator may discontinue or withdraw a participant from the study for the following reasons:

- Pregnancy
- Significant study intervention non-compliance.
- If any clinical adverse event (AE), laboratory abnormality (e.g., elevated LFTs), or other medical condition or situation occurs such that continued participation in the study would not be in the best interest of the participant.
- Disease progression which requires discontinuation of the study intervention
- If the participant meets an exclusion criterion (either newly developed or not previously recognized) that precludes further study participation.
- Participant loses medication.
- Participant lost to follow-up after 3 attempts to contact subject to schedule study visit.
- Participant requests withdrawal of participation.

### Strategies to improve adherence to interventions {11c}

Adherence to protocol will be indicated by attending all visits and taking at least 80% of their medication. Three consecutive daily attempts during the week of the scheduled visit will be made to reschedule a participant’s visit. Medication compliance will be evaluated by counting returned tablets; participants will be asked to bring their bottle of medication at every visit. Participants will also be asked if there was any difficulty in taking the medication during each visit. Adherence will be tracked in a drug accountability log.

### Relevant concomitant care permitted or prohibited during the trial {11d}

Participants receiving concomitant psychotherapy and/or pharmacotherapy for any psychiatric illness will be screened out unless they have been receiving said therapy for at least 3 months before enrolling in the study. This provision is to ensure that previous therapy has stabilized and therefore will not interfere or confound the effect of naltrexone on PGD.

Concomitant use of medications known to cause liver damage, as outlined in the exclusion criteria (section “Criteria for discontinuing or modifying allocated interventions {11b}”), are not allowed to be used during the study due to concern of potentiated liver injury with concurrent use of naltrexone.

### Provisions for post-trial care {30}

If a participant experiences injury from known or unknown risks of the research procedures as described, immediate medical care and treatment, including hospitalization, if necessary, will be available at the usual charge for such treatment.

If the participant screens positive for serious suicidal intent with or without a plan, we will have a licensed mental health professional (e.g., psychiatrist, psychologist) more fully evaluate suicidality and make arrangements for the participant to get to a local hospital emergency room if serious risk is imminent. If not at imminent risk but SI was endorsed, we will refer them to a licensed mental health professional.

No monetary compensation for injury is available.

### Outcomes {12}

#### Primary outcome measures

Difference in PGD symptom severity between the two treatment arms will be measured by using Structured Clinical Interview for PGD (SCIP) and Prolonged Grief-13-Revised (PG-13-R). SCIP will be administered to participants during each clinic visit (weeks 1, 4, and 8). PG-13-R will be administered weekly (weeks 1–12). Given that both these measures are scored, changes in the score from baseline will be analyzed.

#### Secondary outcome measures

Difference in the strength of subjectively perceived closeness of a social relationship between the two treatment arms will be measured by the Inclusion of the other in the Self (IOS) Scale. This scale will be administered at every clinic visit (weeks 1, 4, and 8), as well as at follow-up (week 12). Changes in the numerical strength value from baseline will be analyzed. Measuring social closeness to the deceased is important as we hypothesize that naltrexone will help the bereaved to detach from the deceased. In turn, detachment from the deceased is a necessary first step towards being able to connect with living others.

#### Exploratory outcome measures

Difference in social integration and social connectedness between the two treatment arms will be measured by the Social Integration Questionnaire (SIQ), Interpersonal Support Evaluation List (ISEL), and the Social Connectedness Survey (SCS). All these measures will be administered at every clinic visit (weeks 1, 4, and 8), as well as at follow-up (week 12). Measuring social integration is important as we hypothesize that after being detached form the deceased, participants are better able to socially connect with others. In addition, social support can influence the bereavement process and thus PG-13-R scores.

Differences in SI between the two treatment arms will be measured by the Columbia-Suicide Severity Rating Scale (C-SSRS) at every clinic visit (weeks 1, 4, and 8), as well as at follow-up (week 12). SI is monitored for two reasons: (1) suicide, attempted suicide, and SI have been reported in the post-marketing experience with naltrexone in the use of opioid dependence although no causal relationship has been demonstrated; (2) given that PGD is associated with increased risk of suicide, decreases in PGD symptom severity may correspond to decreased SI.

Differences in health behaviors (e.g., smoking history, alcohol history, health perception, and health-promoting behaviors) between the two treatments arms will be measured by a standard self-reported questionnaire monthly at every clinic visit (weeks 1, 4, and 8), as well as at follow-up (week 12). Bereaved individuals have been shown to exhibit negative changes in health behaviors post-loss [25].

### Participant timeline {13}

**Fig. 1:**
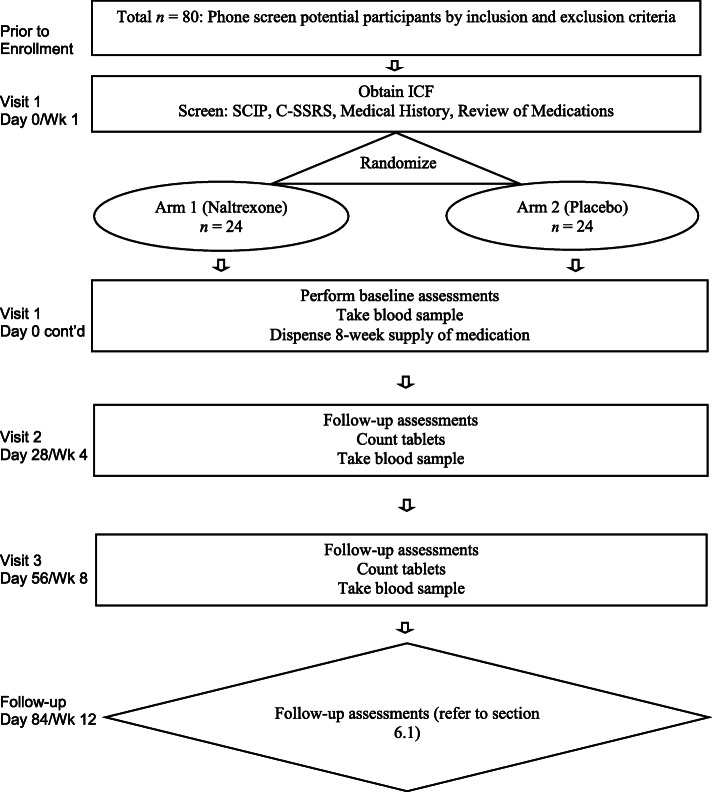
Schema

A schema of this trial is presented in Fig. 1. The total study duration for participants will be 12 weeks. The first 8 weeks are when participants will be taking their assigned medication and attending three clinic visits every 4 weeks. Participants will then be followed up 4 weeks from their last clinic visit.

**Fig. 2:**
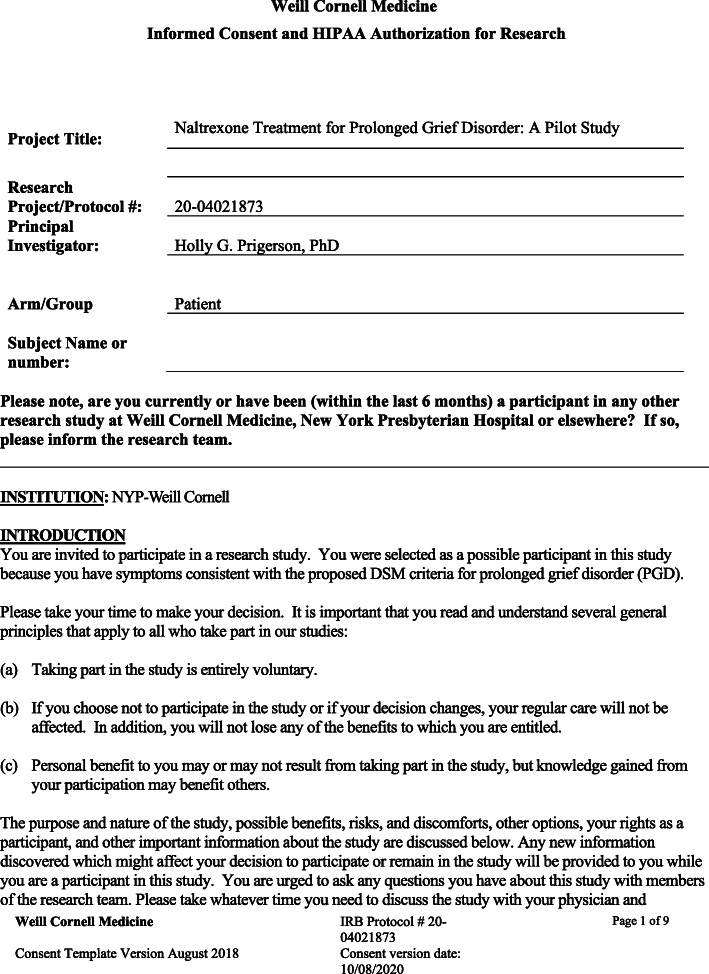
Consent Form

A timeline of assessments is provided in Table 1. Participants will initially be screened over the telephone. If deemed eligible, they will be scheduled for their first clinic visit (day 0) ideally within a week of the telephone screen. During their first clinic visit, participants will be clinically interviewed to determine diagnosis of PGD via SCIP, screened for SI and behavior via the C-SSRS, and verify their past medical history to confirm eligibility. If confirmed eligible, written informed consent will then be obtained (Fig. 2). Participants will then fill out questionnaires regarding their sociodemographic information; current medications; social closeness, integration, and connectedness; and health behaviors. During this visit, participants will be asked to provide a sample of blood and undergo a urine pregnancy test if applicable. At the end of the visit, participants will be dispensed their 8-week supply of medication from the investigational pharmacy.

**Table 1:** Schedule of Assessments

	Pre-study	Week 1	Week 2	Week 3	Week 4	Week 5	Week 6	Week 7	Week 8	Weeks 9–11	Week 12
Phone screen	X										
Randomization		X									
SCIP		X									
C-SSRS		X			X				X		
Medical history		X									
ICF		X									
Sociodemographics		X			X				X		
Medication review		X			X				X		X
IOS Scale		X			X				X		X
SIQ		X			X				X		X
ISEL		X			X				X		X
SCS		X			X				X		X
Health Behaviors Survey		X			X				X		X
Blood sample		X			X				X		
Urine pregnancy test		X			X				X		
Tablet counting					X				X		
Study drug dispensation		X									
Review of side effects					X				X		X
PG-13		X	X	X	X	X	X	X	X	X	X
Adverse event evaluation		X	X	X	X	X	X	X	X	X	X

Participants’ second clinic visit will take place 28 days from their first visit. Like their first visit, participants will be clinically interviewed for PGD, screened for suicide, review any changes to medications, fill out questionnaires, have their blood drawn, and undergo a urine pregnancy test if applicable. Starting from their second visit, participants will be asked about any side effects, and the number of remaining tablets will be counted to determine treatment adherence.

Participants’ third clinic visit will take place 28 days from their second visit and will consist of the same components as their second visit. At the end of this visit, participants will be asked to return any leftover medication.

Follow-up will occur 28 days from their third clinic visit via online assessments that are emailed to participants to complete. These online assessments include a review of side effects; a survey of PGD symptoms; questionnaires regarding social closeness, integration, and connectedness; and health behaviors.

Additionally, during the 12 weeks, participants will be emailed weekly surveys of PGD symptoms.

### Sample size {14}

According to previous studies tracking grief intensity changes across time among the bereaved sample [5, 26], we estimate a natural decline of 1.0 in PG-13-R scores during a period of 2 months with a standard deviation of 6.0, for patients 12 to 18 months post bereavement. Meanwhile, we propose a 20% decline from baseline PG-13-R for the treatment group. The effect size (Cohen’s *d*) was then assumed to be 0.83. The null hypothesis is that comparing to the placebo-controlled group, naltrexone treatment at 50 mg mitigates grief intensity among patients with prolonged grief disorder in a 2-month period. With 48 participants (*n* = 24 naltrexone arm; *n* = 24 placebo arm) and an interim analysis at 50% information fraction, we expect to have 80% power (Type II error < 0.2) to detect a significant difference (Type I error < 0.05) in PG-13-R baseline-to-2-month scores comparing the intervention and control groups. The accrual rate is expected to be 6 participants per month with the recruitment period spanning over 9–12 months.

### Recruitment {15}

We predict an accrual rate of 60%, and therefore to meet the goal of enrolling *N*_total_ = 48, we anticipate a total screening of about 80 individuals. Recruitment methods include referrals, flyers, in-person recruitment, and/or recruitment letters/emails. Recruitment sources include the Cornell Center for Research on End of Life Care website, the department of Geriatrics and Palliative Medicine, the Department of Psychiatry, the Emergency Room, Medical Intensive Care Unit (MICU), local outpatient clinics (e.g., oncology, psychiatry), and local private practices (e.g., psychiatry).

Once IRB approval is obtained, we will list the study as open on the Cornell Center for Research on End-of-Life Care website. We also will post study recruitment fliers with bereavement support groups and relevant clinics at NYP-WCM (e.g., the Wright Center for Aging, the Department of Geriatrics and Palliative Medicine bulletin-board; the Department of Psychiatry bulletin-board, cancer clinic bulletin-boards; Acute Care for Elderly unit). In addition, eligible individuals based on the inclusion criteria may be identified physicians and/or staff of these departments, who will then refer them to our study; individuals may be given an “Agree to Contact” form to complete at this time to allow study staff to contact the participant first. Similarly, co-investigators may approach potential participants in NYP-WCM offices and ask if they would be interested in the study. Trained co-investigators may either screen the participants at recruitment sites and/or ask for their contact information to be followed up at a time convenient for the potential participant. Apart from sources within the NYP-WCM network, we will also contact outpatient psychiatry clinics and private psychiatry practices to make them aware of the study and refer patients.

All potential participants will be logged in a password-protected Excel file stored on a secured, shared server, which will contain their contact information, date of contact, eligibility, recruitment source, and dated of scheduled visit; identifiable health information will NOT be stored during recruitment.

## Assignment of interventions: allocation

### Sequence generation {16a}

The research statistician will use R software to generate a random sequential list of binary codes by participant number. Blocks of fixed length will be applied to keep the number of both arms relatively balanced. Set seed function will be used to ensure that the randomization schedule is reproducible. Participants who have been screened and meet criteria will enter the randomization section and be assigned with the appropriate number and corresponding intervention upon confirmation at the first clinic visit.

### Concealment mechanism {16b}

Only the PI will know what code corresponds to which treatment arm. Trial randomization codes will be kept on a password-protected Excel sheet located on a secure server to which only the PI will have access to.

### Implementation {16c}

Trained research assistants will enroll participants. Once a patient has been successful screened, the research assistant will notify the statistician. The statistician will then generate a binary code that is to be allocated to each participant. Participants will be assigned to interventions based on whichever binary code they are allocated. Neither the statistician nor research assistant will not know which binary code corresponds to which treatment arm.

## Assignment of interventions: blinding

### Who will be blinded {17a}

Because this is a triple-blinded study, all the participants, research staffs, healthcare providers, and statisticians will be blinded to the type of intervention the participant received. This level of blinding is maintained throughout the conduct of the trial, and only when the data has been analyzed according to statistical analysis plan, and conclusions regarding the primary and secondary outcomes are made, will the associated personnel be unblinded.

### Procedure for unblinding if needed {17b}

The principal investigator (PI) at a site may break the blind in the event of an immediate medical emergency (e.g., AE) or abnormal serum laboratory results (e.g., elevation in LFTs), where knowledge of the study participant’s treatment assignment must be known in order to facilitate appropriate medical treatment. In these situations, the investigator must first attempt to contact the study medical monitor before unblinding a participant’s treatment identity in order to obtain concurrence that unblinding a study participant’s treatment assignment is necessary. The one exception to obtaining concurrence with the medical monitor is if a participant tests positive for pregnancy; if this occurs, the PI can and should immediately break the blind.

## Data collection and management

### Plans for assessment and collection of outcomes {18a}

#### Primary outcomes

SCIP is a structured clinical interview is adapted to the DSM criteria for PGD. SCIP will be initially administered at each clinic visit by trained research assistants. Initial administration serves to confirm diagnosis of PGD as well as to establish a baseline. Interviewers will be trained to standard which will be a *κ* > 0.8 agreement between trainee and trainer.

PG-13-R is a self-rated scale consisting of 11 items; for this study, we have added an additional item inquiring about loneliness to be congruent with DSM criteria and SCIP. It was shown that PG-13-R has good internal consistency among three study samples (*α* = .83–.93) [4]. A symptom threshold score of 30 optimized agreement with meeting DSM symptom criteria for PGD (kappa ≥ 0.70 across the datasets) [4]. PG-13-R will be administered in the form of an online survey emailed to participants via REDCap.

#### Secondary outcomes

The IOS is a self-reported pictorial tool that asks respondents to select one of seven pairs of increasingly overlapping circles that best represents their relationship with another, with more overlap signifying a closer relationship. Each pair is numbered, with higher numbers corresponding to stronger perceived closeness. IOS will be initially administered at the first clinic visit, and then every 4 weeks thereafter. This scale possesses good reliability, with (*α* = .93) for the entire sample, (*α* = .87) for family, (*α* = .92) for friendship, and (*α* = .95) for romantic relationships. Test-retest reliability shows similar findings, with (*α* = .83) for the entire sample, (*α* = .85) for family, (*α* = .86) for friendship, and (*α* = .85) for romantic relationships [27]. This scale will be administered by research assistants at each clinic visit, as well as in the form of an interactive online form administered via REDCap.

#### Exploratory outcomes

Social integration and social connectedness will be measured by SIQ, ISEL, and SCS. Each of these measures will be administered by research assistants at each clinic visit, as well as in the form of an online survey emailed to participants via REDCap.

SIQ is a 10-item self-reported scale adapted from The General Social Survey distributed by the National Opinion Research Center. Questions will assess feelings and thoughts while engaged in these activities to gauge the study participant’s level of social integration, and participants will rate the social activities they engage in and the degree of involvement of each.

ISEL is a 40-item self-reported interpersonal support evaluation scale that has been shortened into a 16-item version for this study, as adapted from the Yale Bereavement Study. The participants rate each item regarding how true or false they believe it is on a 4-point scale ranging from “Definitely True” to “Definitely False.”

The SCS is a self-reported survey created for this study consisting of 8 questions that subjectively assess how much participants are socializing, who they are socializing with, and how enjoyable socializing is.

The C-SSRS scale is a scale containing 6 yes-no questions to assess suicidal behavior and ideation. This scale has been proven to have good validity, reliability, sensitivity, specificity, sensitivity to change, and internal consistency for suicidal behavior [28]. At the first clinic visit, the baseline/screening version will be administered a trained research assistant; for all subsequent clinic visits, the “Since Last Visit” version of the C-SSRS will be administered. The “Since Last Visit” version will also be administered as an online survey emailed to participants via REDCap for follow-up.

Health behaviors will be measured by medical outcomes (MOS), health-promoting behaviors (HPB), smoking history (SMO), and alcohol history (ALC). These standard self-reported questionnaires have been used and validated in the Yale Bereavement Study [29]. MOS consists of 11 items that subjectively assesses the recent status of a participants’ health. HPB consists of 15 items that subjectively assess health habits of participants. SMO is administered in two forms. During the first visit, SMO consists of 5 items asking about the participants smoking history and consumption. During the subsequent visits, participants will be asked about current smoking consumption and craving. Likewise, ALC is administered in two forms. During the first visit, participants will be asked 4 questions regarding alcohol history and consumption. During the subsequent visits, participants will be asked about current alcohol consumption and craving.

Side effects will be monitored subjectively and objectively. Subjectively, participants will be asked a nonspecific question (e.g., have you notice any new side effects since your last visit?) at the 2nd and 3rd clinic visit to assess whether any side effects have been experienced since day 1. Objectively, blood samples will be taken by nurses at each clinic visit to be analyzed for AST and ALT, given that the most severe side effect of naltrexone is hepatotoxicity. If female, participants will be asked to take a qualitative urine pregnancy test at each clinic visit, overseen by nurses.

### Plans to promote participant retention and complete follow-up {18b}

Participants will be provided the medication and clinic visits at no cost. Participants will be thoroughly educated about risks of naltrexone, both at the first clinic visit, as well as in the form of a patient handout. All medical procedures (e.g., blood draw, pregnancy test) will be performed by and overseen by trained clinical staff (e.g., nurses). Participants will be encouraged to communicate with research staff via telephone regarding any problems or irregularities. The follow-up visit will be done virtually in the form of emailed surveys for the participants’ convenience.

Adherence to protocol involves attending all visits and taking at least 80% of their medication. Three consecutive daily attempts during the week of the scheduled visit will be made to reschedule a participant’s visit. Medication compliance will be evaluated by counting returned tablets; participants will be asked to bring their bottle of medication at every visit. Participants will also be asked if there was any difficulty in taking the medication during each visit.

Participants who discontinue the study intervention before their 2nd visit will be scheduled for a visit within 7 days of discontinuation to undergo the same procedures of the 3rd visit, and then skip to the follow-up phase 28 days after the hastened visit. Participants who discontinue the study intervention after their 2nd visit but before their 3rd visit will likewise be scheduled for a visit within 7 days of administration to undergo the same procedures of the 3rd visit, and then proceed to the follow-up phase. In addition to the data collected at 3rd visit, data to be collected at the time of study intervention discontinuation will include why the study intervention was discontinued.

### Data management {19}

The data collection plan for this study is to utilize REDCap to capture all treatment, toxicity, efficacy, and adverse event data for all enrolled subjects. REDCap (Research Electronic Data Capture) is a free data management software system that is fully supported by the Weill Cornell Medical Center CTSC. It is a tool for the creation of customized, secure data management systems that include Web-based data-entry forms, reporting tools, and a full array of security features including user and group based privileges, authentication using institution LDAP system, with a full audit trail of data manipulation and export procedures. REDCap is maintained on CTSC-owned servers that are backed up nightly and support encrypted (SSL-based) connections. Nationally, the software is developed, enhanced, and supported through a multi-institutional consortium led by the Vanderbilt University CTSA.

### Confidentiality {27}

Confidentiality will be ensured by removal of identifying information (name, age, gender, race/ethnicity, length of service) of participants and substituting their name with a study participant number. As noted, all data will be de-identified. Nonetheless, we will maintain a file that links subject name with the study participant number thereby enabling us to locate the study participant’s research record if needed. Data will be analyzed in aggregate only, and no identities will be revealed.

Data collected will be obtained by trained and experienced research staff interviewing study participants. The data will be used specifically for the purposes outlined in this proposal and not for any other purpose. All study-related documents will be stored in a secure location until the study has ended and all data analyses are complete. At that time, all study material will be placed in a secured long-term storage facility until it is deemed appropriate to destroy the study material.

### Plans for collection, laboratory evaluation, and storage of biological specimens for genetic or molecular analysis in this trial/future use {33}

During each clinic visit, participants will be asked to provide a blood sample to monitor elevations in ALT and AST as proxies for liver function, given that hepatotoxicity is the most severe side effect of naltrexone warranting an FDA black-box warning. Upon collection, blood samples will be sent to the NYP-WCM Hospital laboratory for analysis. If participants are female, they will also be asked to undergo a quantitative urine pregnancy test, as naltrexone is classified as a class C medication.

## Statistical methods

### Statistical methods for primary and secondary outcomes {20a}

#### Primary outcome

PG-13-R baseline-to-2-month scores for each participant will be calculated as participants’ PG-13-R scores at 2-month follow-up minus the scores at baseline. A two-sample *t*-test comparing the PG-13-R baseline-to-2-month scores between intervention and control groups will be conducted as primary statistical analysis. If the normality assumption for *t*-test is breached, Wilcoxon rank-sum test will be used instead. In addition, multivariate linear regression model will be fitted to adjust for confounders. All statistical tests will be two-sided.

In addition to PG-13-R baseline-to-1 month scores, PG-13-R baseline-to-3 month scores will be calculated separately. A two-sample *t*-test comparing each type of scores between intervention and control groups will be conducted as secondary statistical analyses. If the normality assumption for *t*-test is breached, a Wilcoxon rank-sum test will be used instead. In addition, multivariate linear regression model will be fitted to adjust for confounders.

Simultaneously, a two-sample proportion test will be conducted for SCIP diagnosis at 1 month, 2 months, and 3 months post baseline. If the approximation assumption for the proportional test is breached, Fisher’s exact test will be used instead. In addition, multivariate logistic regression model will be fitted to adjust for confounders. All statistical tests will be two-sided.

#### Secondary outcome

We will first examine bivariate associations (e.g., using Pearson correlation coefficients) between the score on the IOS (in which lower scores will reflect less “integration with other” as represented by the quantified degree of circle overlap) and assignment to naltrexone vs. placebo. We also expect IOS scores to be related to reductions in PG-13-R scores and C-SSRS scores of SI and scores of greater social integration and support (SIQ and ISEL, respectively). Assuming that linear regression analyses indicate that naltrexone is significantly associated with reductions in PG-13-R scores and IOS scores, and that IOS scores are significantly associated with PG-13-R scores, we will then regress PG-13-R scores on naltrexone and IOS scores. If the effect of naltrexone loses statistical significance in relation to PG-13-R scores, then following Baron and Kenney’s method of testing for mediation, it can be concluded that IOS scores (i.e., social integration) is a mediator and the mechanism through which naltrexone operates to reduce PGD symptomatology.

### Interim analyses {21b}

Since this is a small-scale trial, it is unlikely that there is any meaningful discouraging or encouraging primary results prior to the end of the trial. Therefore, no interim analysis will be conducted for the trial as the goal of the study is gathering evidence for efficacy/futility at the end of the study.

### Methods for additional analyses (e.g., subgroup analyses) {20b}

We will conduct a secondary analysis to explore whether the treatment has an effect in a shorter time frame based on weekly PG-13-R surveys, using similar methods described above.

### Methods in analysis to handle protocol non-adherence and any statistical methods to handle missing data {20c}

Missing data may be estimated using a multiple imputation procedure described by Schafer and Olsen [30].

### Plans to give access to the full protocol, participant-level data, and statistical code {31c}

The datasets analyzed during the current study are available from the corresponding author upon request after obtaining approval of the use by all members of the investigative team.

## Oversight and monitoring

### Composition of the coordinating center and trial steering committee {5d}

The trial steering committee is composed of the PI and three co-investigators, who all contributed to and approved the final protocol. The PI is responsible for oversight of the entire study. One co-investigator oversees the daily operations of the study, including recruitment, enrollment, consenting, and the activities involved during clinic visits, with ancillary support from research assistants and nurses. Two co-investigators are psychiatrists who provide clinical support. The steering committee meets monthly to discuss conduct and progress of the study.

### Composition of the data monitoring committee, its role and reporting structure {21a}

Given that risks posed to participants are low, we propose to use an independent medical monitor. This independent medical monitor will be a board-certified psychiatry physician who will not be involved in the study apart from his/her capacity as the medical monitor.

### Adverse event reporting and harms {22}

**Fig. 3:**
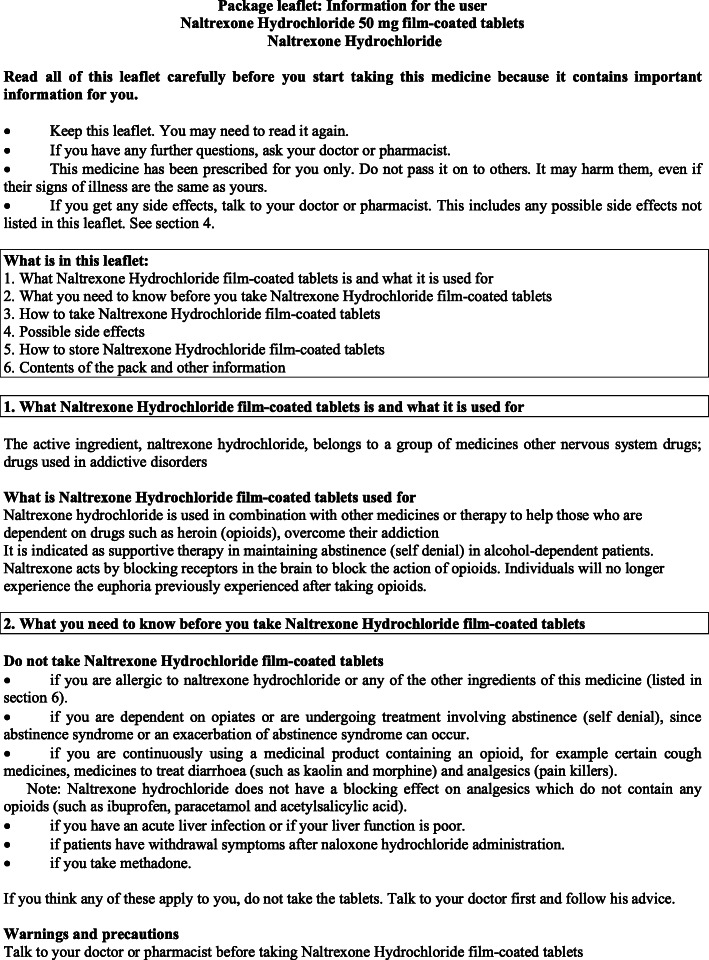
Naltrexone patient information sheet

Blood samples will be collected at each clinic visit to monitor elevations in LFTs, as hepatotoxicity is a known, but rare, severe adverse effect of naltrexone (Fig. 3). Urine pregnancy tests will be administered at each clinic visit, as naltrexone is classified as a category C medication. Participants will also be asked to report any new side effects that they may have experienced after starting the study during visits. Participants will also be able to contact the PI via the phone number listed on the ICF at any time to report adverse effects. All AEs will be reviewed weekly to the independent medical monitor. The medical monitor’s comments/review will be submitted to the IRB at the time of continuing review and submitted to participating sites upon receipt of review comments.

Adverse events that may cause the subject to terminate protocol treatment include an elevation of LFTs twice that of baseline, becoming pregnant, as well as any other adverse effects that significantly affect quality of life.

Study stopping rules are based on efficacy and safety. In regard to safety, the study will be suspended if > 3 participants in either arm are found to have significantly elevated LFTs. The PI will review these adverse events, and if these events are occurring within the naltrexone arm, then the study will be terminated. If these events are found to be occurring within the placebo arm, then those participants affected will be suspended from the study until receiving clearance from a physician.

### Frequency and plans for auditing trial conduct {23}

Given that this is a small-scale trial, there are no plans for auditing trial conduct. The IRB and CTSC have the right to audit studies.

### Plans for communicating important protocol amendments to relevant parties (e.g., trial participants, ethical committees) {25}

Protocol amendments will be submitted to the appropriate ethics committee. All agreed protocol amendments will be clearly recorded on a protocol amendment form and will be signed and dated by the original protocol approving signatories. All protocol amendments will be submitted to the relevant institutional IRB for approval before implementation, as required by local regulations. The only exception will be when the amendment is necessary to eliminate an immediate hazard to the trial participants. In this case, the necessary action will be taken first, with the relevant protocol amendment following shortly thereafter.

## Dissemination plans {31a}

Individual-level de-identified patient data will be made publicly available after the study-specific aims have been published. The statistical analyses will be available for those who request it based on published analyses. Authorship of the final report will be based on contribution to the trial as determined by the principal investigators. The final report will be published in a peer-reviewed journal to facilitate communication to healthcare professionals and the general public. Published results will be shared with study participants should they indicate an interest in receiving this information (e.g., publications of these data will be sent as a pdf to their email address).

## Discussion

This trial is the first of its kind to investigate the efficacy of using oral naltrexone as pharmacological treatment for PGD. The proposed study addresses both a conceptual and practical need regarding the treatment of PGD. Conceptually, the development of treatment for PGD is in its early stages, and studies concerning pharmacological treatment are especially lacking. Practically, patients with PGD are in dire need for a rapid, convenient, safe, and effective intervention to promote their bereavement adjustment while diminishing the substantial risks posed by PGD.

There are two statistical concerns. First, this is a single-site study that may limit its external validity. Second, this study has limited statistical power. If the results of this initial study are promising, however, these data may be used in support of a larger-scale, adequately powered study.

Naltrexone has the theoretical potential to be another form of treatment that can improve the mental health, physical health, and well-being of the bereaved with PGD. If shown to be effective, this trial may serve to change the way PGD is understood, paving the way for further research on the reward system in PGD and bereavement in general.

## Trial status

This manuscript was based on protocol version 2.2, dated 10/08/2020.

Recruitment for this study began on October 21, 2020. Recruitment is expected to be completed by approximately October 31, 2021.

## Data Availability

Any data required to support the protocol will be supplied if all members of the investigative team approve the request.

## Abbreviations

AE: Adverse event
ALC: Alcohol history
ALT: Alanine transaminase
AST: Aspartate transaminase
C-SSRS: Columbia-Suicide Severity Rating Scale
COVID-19: Coronavirus disease 2019
CTSC: Clinical and Translational Science Center
DSM: Diagnostic and Statistical Manual of Mental Disorders
FDA: Food and Drug Administration
HPB: Health-Promoting Behaviors
IOS: Inclusion of the other in the Self Scale
ISEL: Interpersonal Support Evaluation List
MDD: Major depressive disorder
MOS: Medical outcomes
NYP-WCM: NewYork Presbyterian-Weill Cornell Medicine
PG-13-R: Prolonged Grief-13-Revised
PGD: Prolonged grief disorder
PI: Principal investigator
PO: Per os (by mouth)
PTSD: Post-traumatic stress disorder
SCIP: Structured Clinical Interview for Prolonged Grief Disorder
SCS: Social Connectedness Survey
SI: Suicidal ideation
SIQ: Social Integration Questionnaire
SMO: Smoking history
SSRI: Selective serotonin receptor inhibitors
TCA: Tricyclic antidepressants
